# Dr Yeats's Answer to Dr. Lubbock, Relative to Mayow

**Published:** 1799-09

**Authors:** G. D. Yeats

**Affiliations:** Bedford


GENTLEMEN,

AS Dr. LUBBOCK's remarks on my letter require an anſwer, I ſhall be obliged to you to inſert in your next number the following obſervations:

By aſſerting that MAYOW's name was not mentioned in any author whom I had conſulted, publiſhed during his life, I could not poſſibly imply that others whom I had not ſeen did not take notice of him. From the ſilence, however, of many cotemporary authors of the firſt reſpectability, I felt a confidence in concluding, that the doctrines of Mayow, did not meet with that immediate reception to which they were juſtly entitled by the accurate experiments from which they were drawn; and I muſt ſtill withhold my aſſent to any great diffuſion of his opinions during his life. I aſſert not this from prejudice, or from a blindneſs to conviction; for by promoting the inveſtigation, I ſhall feel equal pleaſure, whether the ſpark of truth be ſtruck out by the genius of Dr. Lubbock, or by my own humble exertions. The various reading of Dr. Lubbock has led him to but one author who has mentioned Mayow during his life, and notwithſtanding this author filled a Profeſſor's chair, and not without celebrity, yet either from the want of a genius among his pupils, or, more probably, from the want of a conviction of of the truth of Mayow's doctrines, they did not acquire that publicity, which a minute detail, before a numerous audience, would readily have produced; for, had ETMULLER fully comprehended the extent and importance of Mayow's diſcoveries, he would not have failed to have related them to his pupils with all the impreſſive force of a profeſſor, and their young minds would certainly have yielded to the impreſſion in ſuch a manner, as to give to the doctrines an immediate and extenſive circulation. Such an intereſting curioſity would have been excited, that no phyſiologiſt would have appeared before the public, without paſſing ſome obſervations on a topic which engaged ſo general an attention; but it was far otherwiſe, for the doctrines of STAHL prevailed by the celebrity of their author, over the infancy of Mayow's diſcoveries: but ſevere, no doubt, would have been the ſtruggle, had Mayow's life been ſpared. With reſpect to MUNDAY, I am ſupported in my opinion by Dr. SCHERER, of Weimar, in Germany. This ingenious chemiſt has juſt publiſhed an edition of Mayow's works, tranſlated into the German language, with a copy of which he has favoured me; it is to be followed by a commentary.

From the merits of LOWER, I by no means wiſh to detract. He was one of the firſt who pointed out the difference between arterial and venous blood, and the cauſe of that difference; for in the preface to the fourth edition of his treatiſe on the heart, he obſerves, "Nemo hactenus venoſi atque arterioſi (ſanguinis) quoad colorem diſcrimen clare illuſtrandum ſuſceperit." No experiments ſo clearly proved and illuſtrated this ſubject as thoſe of the indefatigable Mayow; and, notwithſtanding the fourth edition of the Tractatus de corde, was publiſhed (1680) twelve years after Mayow's Treatiſe on Reſpiration and the Rickets (1668), and of which an account was given in Phil. Tranſ. of that year, and ſix years after the complete edition of his works (1674), yet not a ſingle obſervation is to be found concerning Mayow or his doctrines. Mayow does not betray ſuch illiberality towards Lower, whoſe experiments on the difference of colour in the blood he quotes in chap. viii. p. 148 of his works:—a proof that Lower muſt have written previous, at leaſt, to the publication of this part of Mayow's works.

I muſt alſo ſtill be of the opinion that THRUSTON ought to have quoted Mayow.—Becauſe a friend writes a letter of congratulation to an author on the publication of a particular doctrine, it is no proof of the difference of ſuch a doctrine, and upon no account could Mayow quote opinions that were merely whiſpered in converſation, particularly as it was impoſſible for him to vouch for their authenticity. Others, too, might have diſputed with Thruſton the priority of right to the promulgation of ſuch doctrines, which indeed is rendered rendered probable from what Thruſton himſelf ſays, and of which Dr. Lubbock has taken notice.—In page 106 of Obſervations on Modern Diſcoveries, I have ſaid, when ſpeaking of Thruſton's opinions, "How ſimilar, with ſome exception, is this to the reaſoning of Mayow. Were the experiments of Mayow, before publication, noiſed abroad, ſo as to ſuggeſt theſe ideas to Thruſton, or did opinions, like theſe, beginning to prevail at that time, from obſervation, rouſe the genius of Mayow to inſtitute experiments to confirm the truth of them?" It is certainly very remarkable, that experiments ſo well conducted as thoſe of Mayow, and inſtituted with a view of illuſtrating a point ſo much the ſubject of inveſtigation at that time, ſhould not have received the honour of quotation from ſuch men as Thruſton and Lower.

The crude ſtate in which Mayow found chemical ſcience, obliged him to invent terms, the ambiguity of which perplexed many, but ſtill, however, he made a very great diſtinction between nitre and nitro-aëreal particles, and was ſolicitous on all occaſions, that his readers ſhould make the ſame diſcrimination. He ſets out with giving, in ſome degree, a nomenclature, and becauſe he found in nitre active particles, ſimilar to the active part of atmoſpheric air, he terms them as being common to both, "particulæ anitro aëreæ. Quocirca particulas iſtas igneas nitro aërique communes, particulas nitro-aëreas, ſive ſpiritum nitro-aëreum in futurum nuncupare liceat," p. 18. He ſeems anxious to avoid the expreſſion nitrum aëris, leaſt he ſhould convey the idea that nitre, as ſuch, was contained in the atmoſphere. Surely I have good reaſons for concluding that ſuch was the opinion of ſome of the authors quoted by Dr. Lubbock, when in one ſhort paſſage taken by him from Connor, the words nitrum aëris are repeatedly mentioned.

In p. 242 of Obſervations on Modern Diſcoveries, the following remarks on Mayow's terms will be found :—"The equivocal expreſſions, particulæ nitro-aëreæ, nitro-ſalinæ, &c. which Mayow uſed, is a reaſon why he was ſo eaſily miſunderſtood by thoſe who did not thoroughly examine into his expreſſions, and alſo afforded an opportunity for others to aſcribe to him opinions which he never publicly avowed. Science was not ſufficiently improved to allow of better, and as far as chemiſtry was advanced at this period, no terms could be more aptly applied. Had Mayow lived to an older age, his terms would no doubt have been altered; as by a variety of experiments, he would have extended his diſcovery of oxygen more generally, and have ſeen the neceſſity of inventing expreſſions leſs equivocal."

Having thus finiſhed my anſwer to Dr. Lubbock, whoſe candour in the controverſy claims my acknowledgments, permit me, gentlemen, to make a few more obſervations reſpecting Mayow.

Any

Any one who has ever read the Tractatus quinque of Mayow, muſt have diſcovered a depth of penetration and an accuracy of obſervation rarely to be met with in ſo young a man. In him were combined many requiſites for a great literary character, a perſeverance in reſearch, with a judgment for its direction. It is a little extraordinary, that FOURCROY ſhould charge Mayow with not having puſhed his diſcoveries farther, and that he did but little more than enter upon a career, of the extent of which he had no conception, and that he was not ſufficiently ſtruck with the importance of his diſcoveries. The French chemiſt has, in other inſtances, done ample juſtice to our countryman, but in the above aſſertion he does not ſeem to have applied his uſual accuracy. Let it be remembered, that the career, upon which Mayow entered terminated only with his life, and no obſtacles, however great, checked the ardour of his purſuits. The illiberal and unphiloſophical ſilence obſerved towards him, in ſubjects too, upon which his labours had thrown the greateſt light, rouſed not his reſentment, and the imputation of novelty in his doctrines, perhaps of youth in himſelf, reſtrained not that noble ſpirit of inquiry with which he was endowed. I am not, I think, too ſanguine in imagining, that had Mayow lived to an advanced age, his ardent mind would not have ſuffered doctrines to have been loſt in oblivion for the want of reiterated and varied experiments to confirm the truth of them; and he would have anticipated in a great meaſure that noble ſyſtem of chemiſtry which has ſo admirably refuted the doctrine of phlogiſton; and ſo fully convinced was he of the truth of thoſe opinions which he adopted, that in the dedication of his works to Mr. COVENTRY, he makes the following dignified and manly exordium. " Tractatus hoſce nomini tuo, vir clariſſime, non eo animo inſcripſimus, ut eorum patrocinium & defenſionem ſuſciperes: veritas enim ipſa ſe defendet; et ſi quid erroris, pro humani generis infelicitate admiſſum ſit, patronum ei quærere non debeo, cui hoſtem me eſſe cum primis profiteor." Mayow certainly did not confine the inferences from his experiments within a narrow compaſs, as the chapters, De motu muſculari, De ſpiritibus animalibus, and other parts of his works, fully evince. He endeavoured to explain the laws of the animal economy from his chemical diſcoveries, and he certainly has a fair claim in many inſtances to ſucceſsful conjecture, and in moſt, to more than plauſible theory. He was indeed the LAVOISIER of the ſeventeenth century. I beg to be underſtood as ſpeaking relatively to the ſtate of the ſciences at the different periods in which theſe great men lived.

Before his death Mayow repaired to London (at what period I cannot exactly ſay), probably with a view of exerting thoſe abilities in the metropolis, which had been ſo ſucceſsfully employed in provincial practice. He was was, no doubt, ambitious of being confpicuous in the Royal Society, and alfo by farther elucidation, of confirming his chemical difcoveries.—He died at the houfe of an apothecary in York-ftreet, near Covent Garden, his conftitution having probably been injured by conftant and fevere ftudy, which an unfortunate marriage was ill-calculated to repair.

I am, Gentlemen,

Yours, &c.

Bedford, *Aug*. 8, 1799. G. D. YEATS.

---

### *To the Editors of the Medical and Phyfical Journal.*

Gentlemen,

